# A Diagnostic Dilemma of Bleeding Gastric Tumor or Bleeding Pancreatic Tumor

**DOI:** 10.7759/cureus.13068

**Published:** 2021-02-01

**Authors:** Oseen Shaikh, Suresh Chilaka, Gopal Balasubramanian, Uday Kumbhar, Muhamed Tajudeen

**Affiliations:** 1 Surgery, Jawaharlal Institute of Postgraduate Medical Education and Research, Puducherry, IND

**Keywords:** gist, pancreas, stomach, laparoscopy

## Abstract

Gastrointestinal stromal tumors are the most common nonepithelial tumors of the gastrointestinal tract. The stomach is the most common site of occurrence. Most of the tumors are asymptomatic. Many patients may present with mass per abdomen, gastrointestinal bleed. Tumors arising from the stomach’s posterior wall may grow large, and on imaging, it may create confusion of the site of origin. We present a case of gastrointestinal stromal tumor arising from the stomach’s posterior wall, growing large and creating a confusion of site of origin.

## Introduction

Gastrointestinal stromal tumors (GISTs) are the most common mesenchymal tumors of gastrointestinal tracts. The exact incidence of GIST was initially unclear, but recent studies showed that the incidence ranged from seven to nine cases per million people [1]. Middle and older age group people are most commonly affected [2]. GIST is usually asymptomatic and usually detected incidentally during imaging studies or endoscopy and performed for other indications. Some patients may have mild pain, bloating, or dyspepsia. Few patients with large GIST may present with a palpable mass or compressive symptoms. Few present with acute gastrointestinal bleed that may require urgent endoscopic, surgical or radiological intervention [3-5]. In this case report, we present a patient presented with acute upper gastrointestinal bleed features, later diagnosed with gastric GIST, managed with surgical intervention.

## Case presentation

A 29-year-old male patient, without any significant past medical or surgical history, presented with hematemesis complaints about three days, coffee ground color, and sometimes bright red color containing clots. He also had a history of melena for the past three days. The patient did not have any history of abdominal pain or history suggestive of liver disease. He was non-alcoholic and non-smoker. The patient had presented to a local hospital, where blood transfusion was done, and he was referred to our hospital for further management. On presentation patient was dehydrated, severe pallor and tachycardia were present. Initially, the patient was managed conservatively with intravenous fluids and blood transfusion.

**Figure 1:**
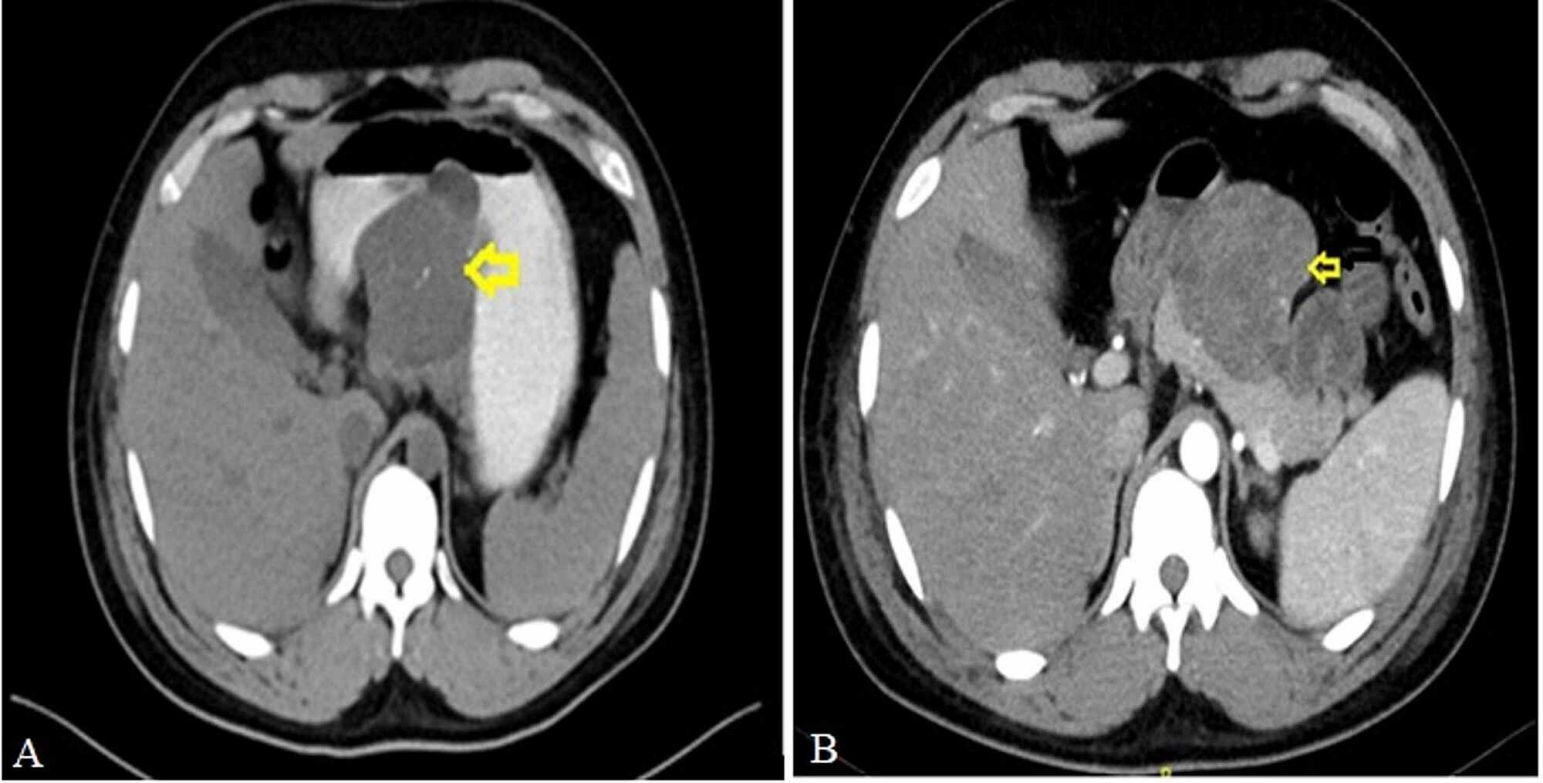
Computed tomography abdomen showing tumor in the lesser sac (arrow) involving posterior wall of stomach and pancreas. (A) With oral contrast. (B) Without oral contrast.

Upper gastrointestinal endoscopy was done, which showed normal mucosa of the stomach with a bulge along the stomach’s greater curvature. The site of bleeding could not be visualized. Contrast-enhanced computed tomography (CECT) abdomen with oral contrast was done, showing a large 8 × 6 × 7 cm predominantly exophytic mass involving posterior wall body and greater curvature and lesser curvature of the stomach with infiltration to the pancreas (Figure 1).

However, imaging was not definitive, whether the tumor was arising from the stomach or the pancreas. Endoscopic ultrasonography (EUS) was done for definitive diagnosis. During EUS, it was observed that the stomach was full of blood clots; hence the procedure was deferred, and the patient was planned for surgery.

Based on the presentation and investigation done, we had thought of three differential diagnoses. Gastric GIST, which is infiltrating into the pancreas, was our first diagnosis, supported by the patient’s age and clinical symptoms. Although very rare, pancreatic GIST is infiltrating into the stomach, but it was our second differential diagnosis. Solid tumor of the pancreas presenting as acute gastrointestinal bleed was our third differential diagnosis, which is also rare, as these tumors are common in females and occur in significantly a younger age group.

**Figure 2:**
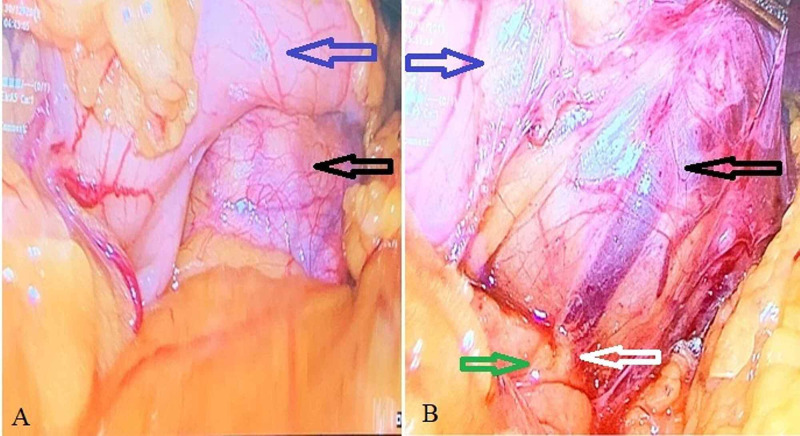
Intraoperative images showing the laparoscopic view. (A) A tumor (black arrow) arising from the posterior wall of the stomach (blue arrow). (B) Clear fat plane (white arrow) maintained between tumor and pancreas (green arrow).

Diagnostic laparoscopy was done. There was no evidence of free fluid in the abdomen, liver, or peritoneal metastasis. Lesser sac was opened, there was an exophytic growth arising from the posterior wall of the stomach extending towards the lesser curvature. The tumor was abutting the anterior surface of the head and body of the pancreas. There was no infiltration into the pancreas (Figure 2).

**Figure 3:**
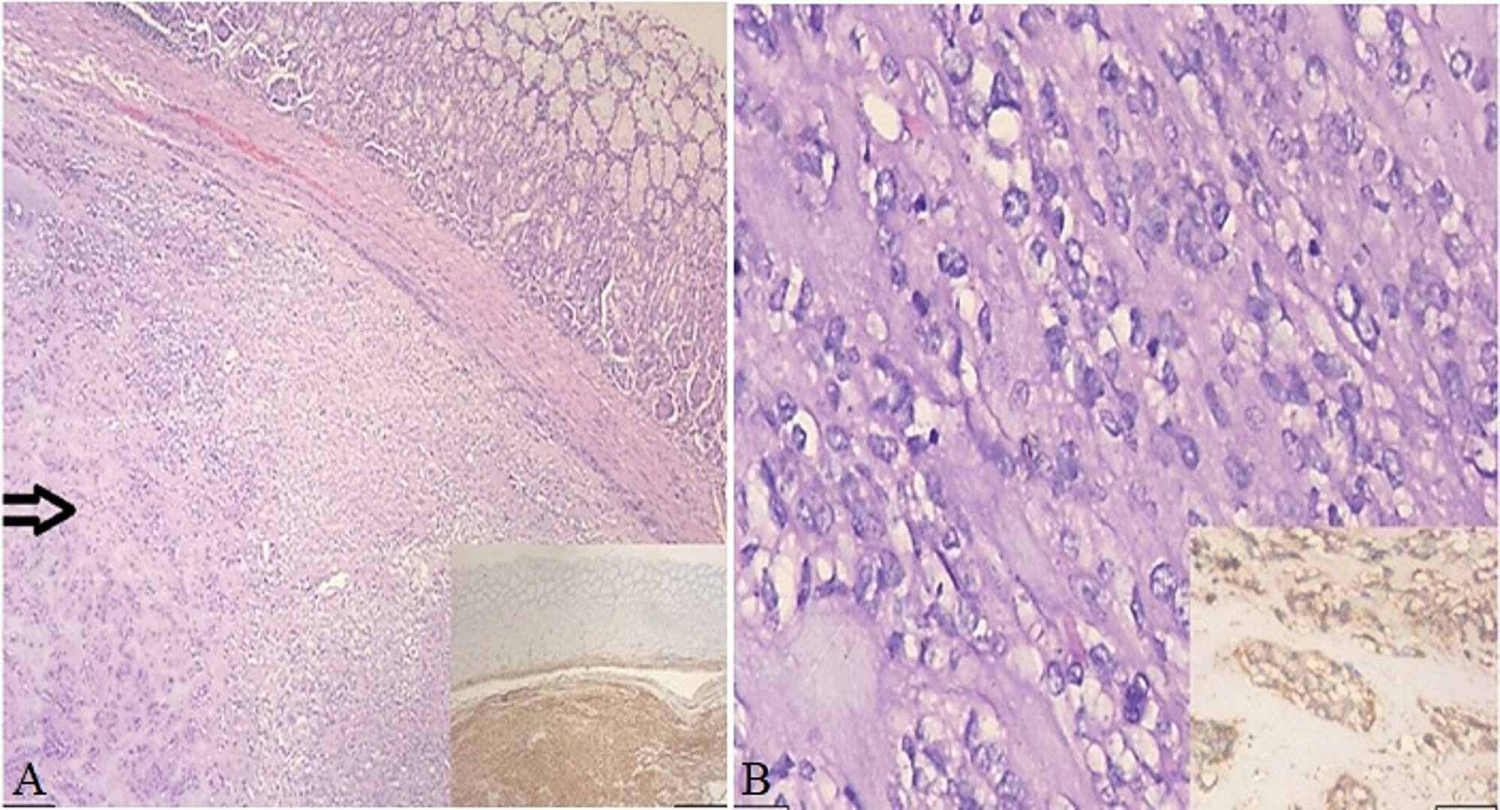
Histopathological image of the gastrointestinal stromal tumor. (A) Gastric mucosa with underlying submucosa and muscularis propria entirely replaced by a tumor (arrow) with varying cellularity and epithelioid cell morphology with discovered on gastrointestinal stromal tumor (DOG1) positive immunohistochemical staining (right lower corner). (B) Higher magnification of the tumor with epithelioid morphology of the tumor cells and brisk mitotic activity with a cluster of differentiation (CD117) positive immunohistochemical staining (right lower corner).

Wedge resection of the tumor with a 2-cm margin and gastro-gastrostomy was done. The postoperative period was uneventful without any complications. The patient improved well, discharged after a week, and followed up for a period of one year. The specimen was sent for histopathological examination, and features were consistent with GIST (Figure 3).

## Discussion

A GIST is the most common mesenchymal tumor of the gastrointestinal tract. The most common site is the stomach (56%), followed by the small intestine (32%), colon and rectum (6%), and esophagus (<1%) [1]. GIST is known to occur at extra-intestinal sites such as the mesentery, pelvis, pancreas, liver, omentum, and genitourinary tract.

These are known to originate from the interstitial cells of Cajal. There are three different variants of GIST. These can be spindle cell, epithelioid, and mixed. Rarely there may be myxoid stroma, neuroendocrine features, signet ring variant, or marked lymphocytic infiltrate. They are usually positive for cluster of differentiation 117 (CD117) and platelet-derived growth factor-alpha (PDGFA). In patients where these two are negative, discovered on GIST (DOG1) is used as a marker of GIST.

GISTs are usually asymptomatic and are detected incidentally. Others usually will have nonspecific symptoms. Few patients present with a palpable mass or compressive symptoms to adjacent organs. Rarely patients may present with acute gastrointestinal bleed or perforation peritonitis.

Diagnosis is made by imaging and endoscopy. Benign GIST in the stomach may have calcification, which can be seen on plain abdominal radiography. Computed tomography (CT) scan is the investigation of choice. Gastric GIST can be of variable size and may range from few millimeters to centimeters and sometimes very large. Large tumors are usually hypervascular masses and are often heterogeneous because of necrosis, hemorrhage, or cystic degeneration. They often displace but rarely invade adjacent organs. Tumors that are more than 2 cm contain ulceration areas giving a bull’s eye or target lesions. GIST, which gets ulcerated, may present with significant upper gastrointestinal bleeding. Selective embolization of the bleeding vessel can be done if necessary [6]. Although most benign GIST in the stomach has a typical submucosal appearance, tumors that grow outward from the stomach may be challenging to differentiate from extrinsic mass lesions [7]. Our patient CT scan revealed a large mass in the lesser sac with loss of fat plane with the body of the pancreas, creating confusion whether the mass is arising from pancreas or stomach, leading to diagnostic dilemma.

Endoscopy usually shows a submucosal mass, which is smooth in appearance and appears as a stomach bulge. Ulceration may present in a few. We could see the submucosal bulge with normal mucosa with a doubtful focal area of ulceration in our patient. Considering the equivocal and confusion on findings of the CT, the patient was planned for endoscopic ultrasound. We could see that the stomach was full of blood clots during the endoscopy, and hence we could not do the EUS. A biopsy is not needed for diagnosis unless the patient was planned for neoadjuvant chemotherapy or palliative therapy.

Surgery is the main line of treatment for the GIST. Resection with a 1-cm to 2-cm margin with maximum organ preservation is the goal. Systemic chemotherapy should be given to the patient as these are known for recurrence, even after complete resection. Genotyping should be done before starting systemic therapy. Imatinib is the drug of choice for GIST. If the tumor is imatinib resistant, then sunitinib or regorafenib can be tried. Systemic therapy can be given in a neoadjuvant setting whenever the disease is locally advanced, the size of the tumor is large, or tumors at difficult anatomic sites. Our patient had undergone wedge resection of the stomach with gastrostomy. As the patient had intermediate risk based on the histopathology, he was planned for adjuvant treatment. There is no role of radiotherapy in GIST as these are resistant to radiotherapy. However, radiotherapy has been used as palliation for bone metastasis.

## Conclusions

The GIST is the most common nonepithelial tumors of the gastrointestinal tract. Posterior wall gastric GIST may create a diagnostic dilemma when it is large and gives an appearance of pancreatic infiltration. Treatment is mainly surgical. Resection with free margins of the tumor is the only curative option. Laparoscopy plays a significant role in the identification of the tumor origin. Imatinib is used as an adjuvant treatment in moderate and high-risk cases, neoadjuvant treatment in large tumors, and palliative treatment in unresectable cases.
